# Supplementary material for: Monitoring of Avian Influenza Viruses and Paramyxoviruses in Ponds of Moscow and the Moscow Region
Source: Viruses. 2022 Nov 24;14(12):2624. doi: 10.3390/v14122624 (PMC9781285; doi:10.3390/v14122624)
Supplement: Supplementary file 1 [file viruses-14-02624-s001.zip › Supplementary materials/table S1. Full names, designations and accession number of genes of AIV isolated in Moscow in 2006-2019.docx]

| **Designation** | **Isolate Name** | **Subtype** | **Sequence Accession** | **Isolation data** |
| --- | --- | --- | --- | --- |
| g/3100/06 | A/gull/Moscow/3100/2006 | H6N2 | EU152234 - EU152241 | 2006 |
| d/3556/08 | A/duck/Moscow/3556/2008 | H3N1 | MN692225-MN692232 | 13.10.2008 |
| d/3641/08 | A/duck/Moscow/3641/2008 | H11N9 | MT773558 - MT773564, GU991377 | 04.11.2008 |
| d/3661/08 | A/duck/Moscow/3661/2008 | H4N6 | MF680290 - MF680297 | 21.10.2008 |
| d/3720/09 | A/duck/Moscow/3720/2009 | H6N2 | MW269601-MW269607, CY120771 | 21.09.2009 |
| d/3735/09 | A/duck/Moscow/3735/2009 | H4N6 | MF422091-MF422097, CY120772 | 30.09.2009 |
| d/3740/09 | A/duck/Moscow/3740/2009 | H4N6 | MF422098-MF422104, CY120773 | 26.08.2009 |
| d/3799/09 | A/duck/Moscow/3799/2009 | H4N6 | MF422105-MF422111, CY120774 | 04.09.2009 |
| d/3806/09 | A/duck/Moscow/3806/2009 | H3N8 | MN692266 - MN692273 | 04.09.2009 |
| d/4031/10 | A/duck/Moscow/4031/2010 | H6N2 | MT773263 - MT773270 | 20.09.2010 |
| d/4182/10 | A/duck/Moscow/4182/2010 | H5N3 | KF885672 - KF885679 | 16.11.2010 |
| d/4203/10 | A/duck/Moscow/4203/2010 | H3N8 | MN700132 - MN700139 | 26.11.2010 |
| d/4238/10 | A/duck/Moscow/4238/2010 | H3N6 | MN700140 - MN700147 | 16.10.2010 |
| d/4242/10 | A/duck/Moscow/4242/2010 | H3N8 | MN700148 - MN700155 | 13.11.2010 |
| d/4298/10 | A/duck/Moscow/4298/2010 | H3N8 | MN700156 - MN700163 | 22.10.2010 |
| d/4494/11 | A/duck/Moscow/4494/2011 | H3N8 | MN759696 - MN759703 | 27.09.2011 |
| d/4518/11 | A/duck/Moscow/4518/2011 | H4N6 | MF673524 - MF673531 | 04.10.2011 |
| d/4524/11 | A/duck/Moscow/4524/2011 | H3N2+N8 (mix) | MN692213 – MN692221 | 04.10.2011 |
| d/4528/11 | A/duck/Moscow/4528/2011 | H4N6 | MF673532 - MF673539 | 04.10.2011 |
| d/4641/11 | A/duck/Moscow/4641/2011 | H4N6 | MF422112 - MF422119 | 19.10.2011 |
| d/4643/11 | A/duck/Moscow/4643/2011 | H4N6 | KX509943 - KX509950 | 19.10.2011 |
| d/4652/11 | A/duck/Moscow/4652/2011 | H4N6 | KX518711-KX518718 | 11.10.2011 |
| d/4661/11 | A/duck/Moscow/4661/2011 | H3N8 | MN759712 - MN759719 | 11.10.2011 |
| d/4681/11 | A/duck/Moscow/4681/2011 | H3N8 | MN759704 - MN759711 | 21.10.2011 |
| d/4771/12 | A/duck/Moscow/4771/2012 | H4N6 | MF673540 - MF673547 | 10.10.2012 |
| d/4780/12 | A/duck/Moscow/4780/2012 | H3N8 | MT773360 - MT773367 | 31.10.2012 |
| d/4781/12 | A/duck/Moscow/4781/2012 | H4N6 | KX530510 - KX530517 | 31.10.2012 |
| d/4788/12 | A/duck/Moscow/4788/2012 | H3N8 | MT773383 - MT773390 | 17.10.2012 |
| d/4843/12 | A/duck/Moscow/4843/2012 | H4N6 | MF673548 - MF673555 | 17.10.2012 |
| d/4952/13 | A/duck/Moscow/4952/2013 | H5N3 | MN588194 - MN588201 | 26.11.2013 |
| d/4970/13 | A/duck/Moscow/4970/2013 | H1N1 | MN400364 - MN400371 | 26.11.2013 |
| d/4971/13 | A/duck/Moscow/4971/2013 | H5N3 | MN588283 - MN588290 | 19.11.2013 |
| d/5037/14 | A/duck/Moscow/5037/2014 | H3N8 | MT773420 - MT773427 | 26.10.2014 |
| d/5586/18 | A/duck/Moscow/5586/2018 | H1N2 | MN435632 - MN435639 | 17.10.2018 |
| d/5662/18 | A/duck/Moscow/5662/2018 | H1N2 | MN588291 - MN588298 | 01.11.2018 |
| d/5712/19 | A/duck/Moscow/5712U/2019 | H11N6 | MW186784-MW186791 | 21.10.2019 |
| d/5743/19 | A/duck/Moscow/5743/2019 | H1N1 | MW186793-MW186800 | 15.10.2019 |
| d/5744/19 | A/duck/Moscow/5744/2019 | H1N1 | MW186801-MW186808 | 15.10.2019 |
| d/5163/15 | A/ duck/Moscow /5163/2015 | H3N6 | OP132933-OP132940 | 04.09.2015 |
| d/5169/15 | A/ duck/Moscow /5169/2015 | H3N6 | OP133010-OP133017 | 16.09.2015 |
| d/5171/15 | A/ duck/Moscow /5171/2015 | H3N6 | OP133377-OP133384 | 07.09.2015 |
| d/5172/15 | A/ duck/Moscow /5172/2015 | H3N6 | OP133387-OP133394 | 07.09.2015 |
| d/5881/21 | A/ duck/Moscow /5881/2021 | H3N2 | OP133621-OP133628 | 06.10.2015 |
| d/5897/21 | A/duck/Chernogolovka/5897/2021 | H3N8 | OP136008-OP136015 | 07.11.2021 |
| d/5908/21 | A/duck/Chernogolovka/5908/2021 | H3N8 | OP135948-OP135955 | 07.11.2021 |
