# Supplementary figures and images for: Monitoring of Avian Influenza Viruses and Paramyxoviruses in Ponds of Moscow and the Moscow Region
Source: Viruses. 2022 Nov 24;14(12):2624. doi: 10.3390/v14122624 (PMC9781285; doi:10.3390/v14122624)

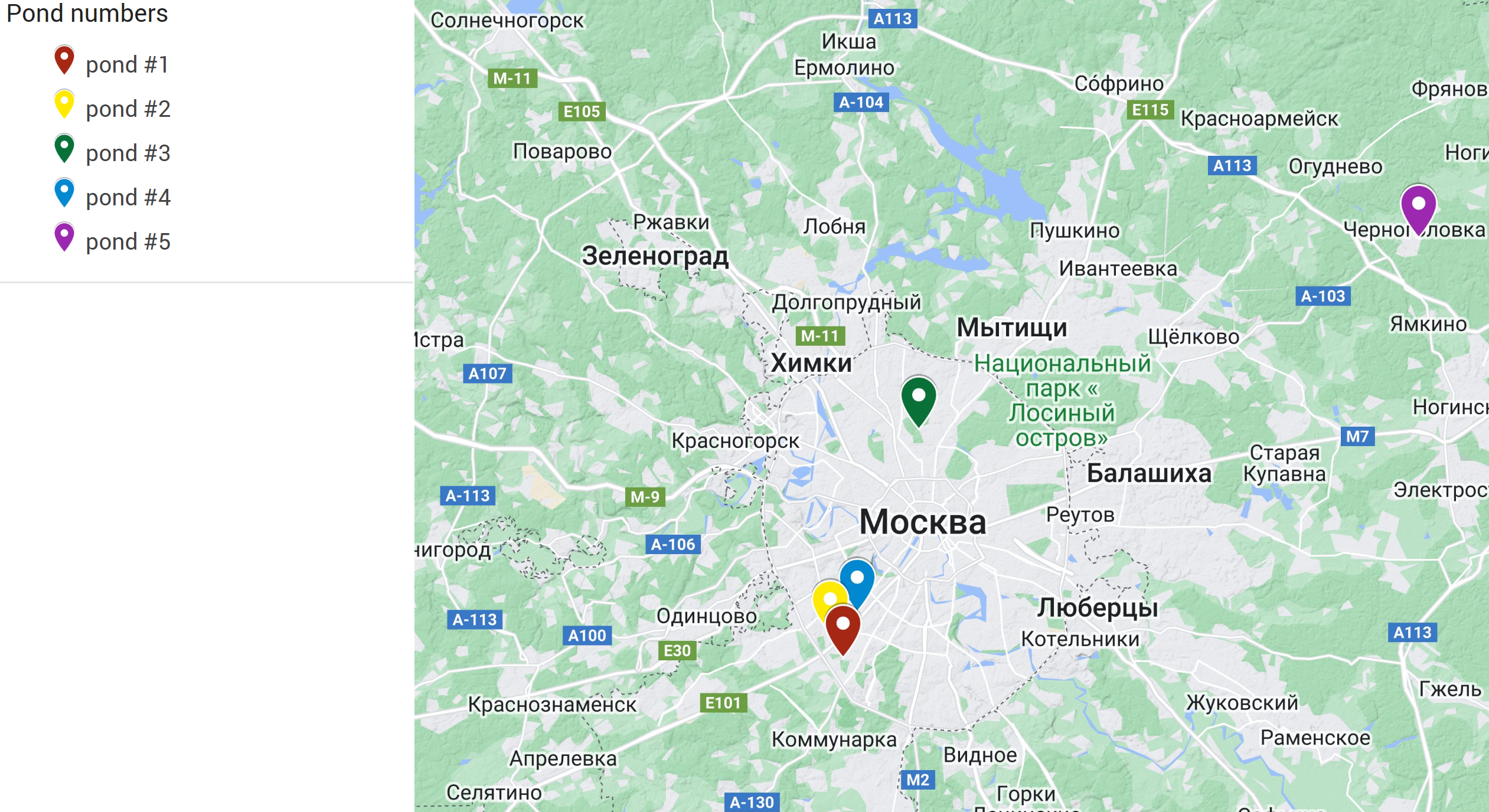

Supplement: Supplementary file 1 [file viruses-14-02624-s001.zip › Supplementary materials/Figure S1 Map of the sampled ponds location.png]

Tree scale: 0.1

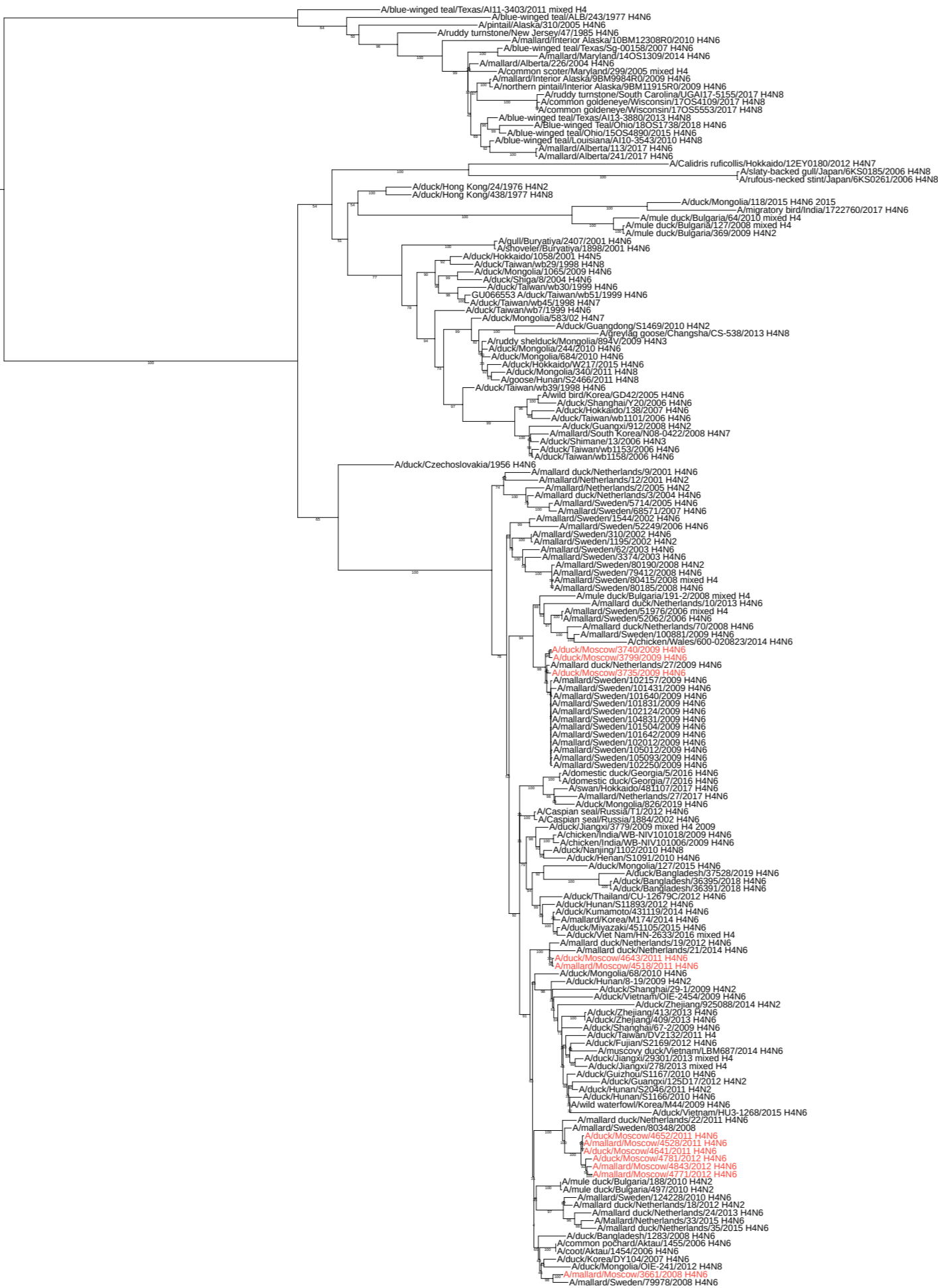

Supplement: Supplementary file 1 [file viruses-14-02624-s001.zip › Supplementary materials/figure S10 evolutionary tree of the H4 HA gene.pdf]

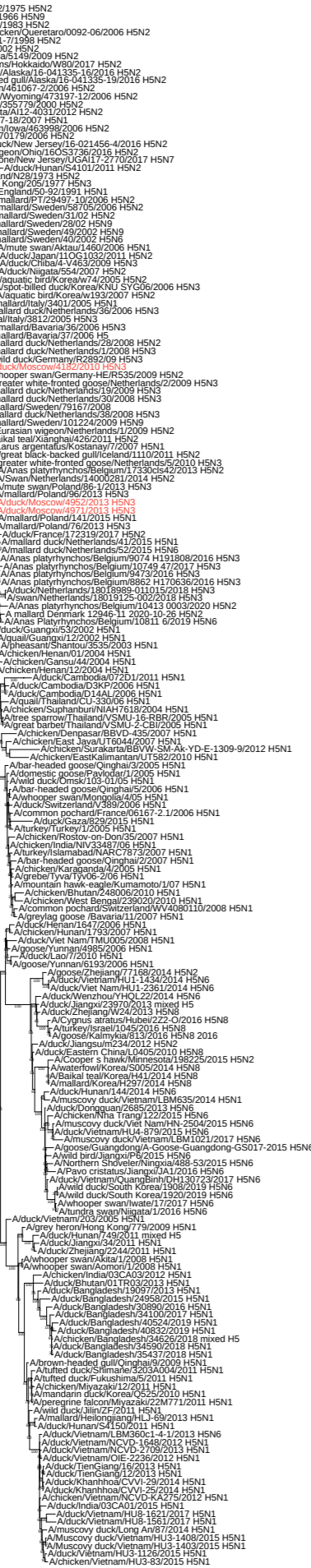

Supplement: Supplementary file 1 [file viruses-14-02624-s001.zip › Supplementary materials/figure S11 evolutionary tree of the H5 HA gene.pdf]

Tree scale: 0.1

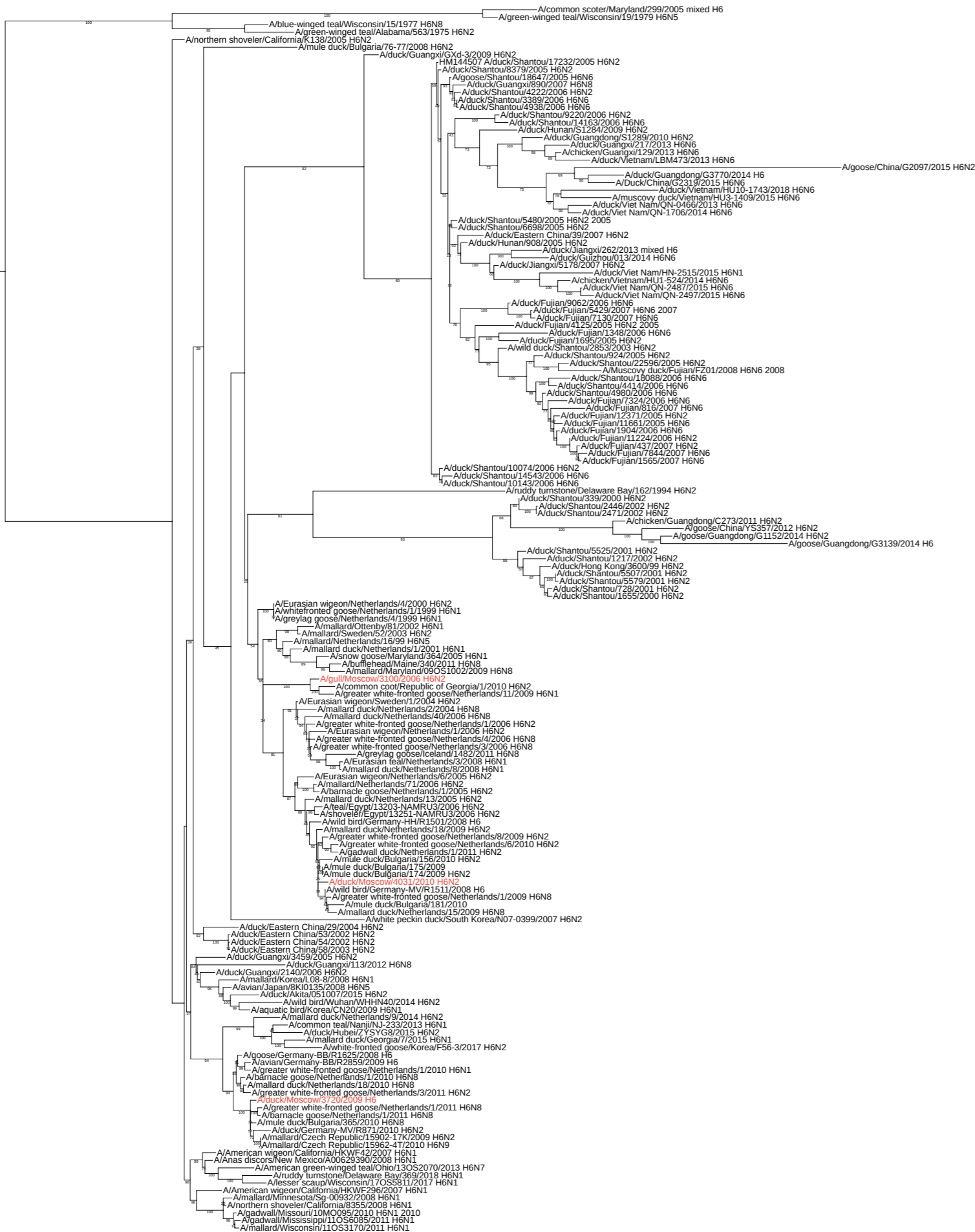

Supplement: Supplementary file 1 [file viruses-14-02624-s001.zip › Supplementary materials/figure S12 evolutionary tree of the H6 HA gene.pdf]

Tree scale: 0.1

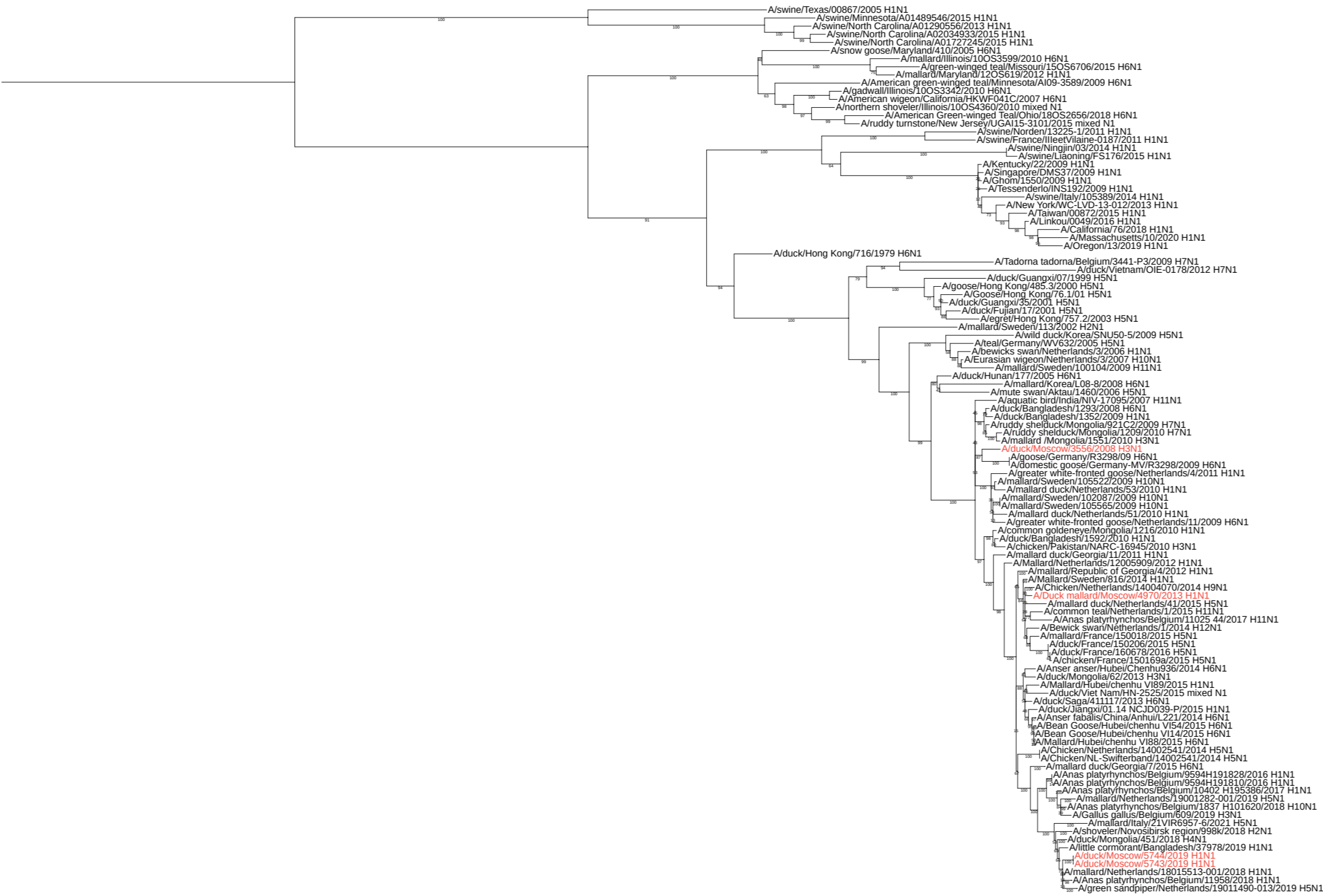

Supplement: Supplementary file 1 [file viruses-14-02624-s001.zip › Supplementary materials/figure S14 evolutionary tree of the N1 NA gene.pdf]

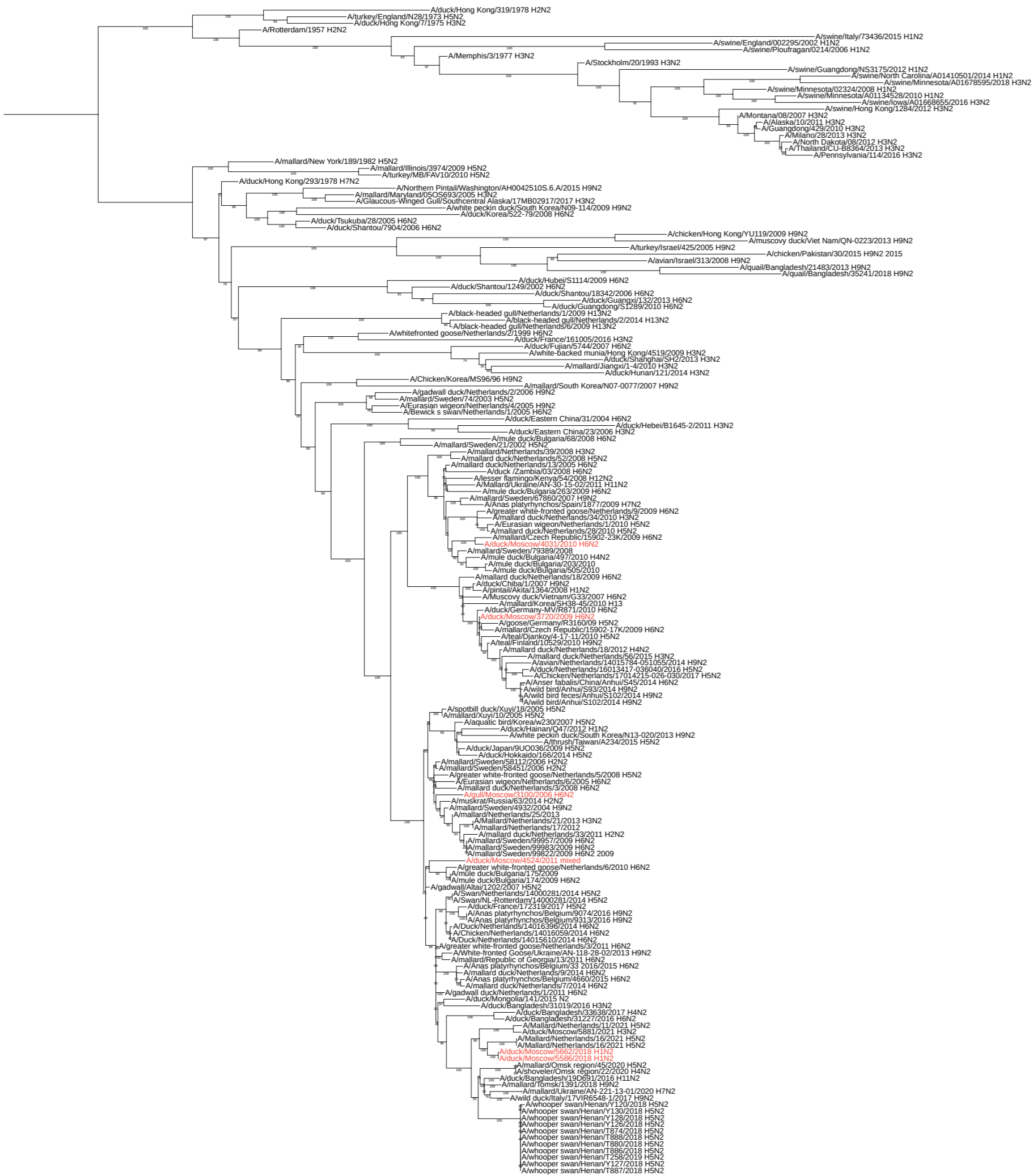

Supplement: Supplementary file 1 [file viruses-14-02624-s001.zip › Supplementary materials/figure S15 evolutionary tree of the N2 NA gene.pdf]

Tree scale: 0.1

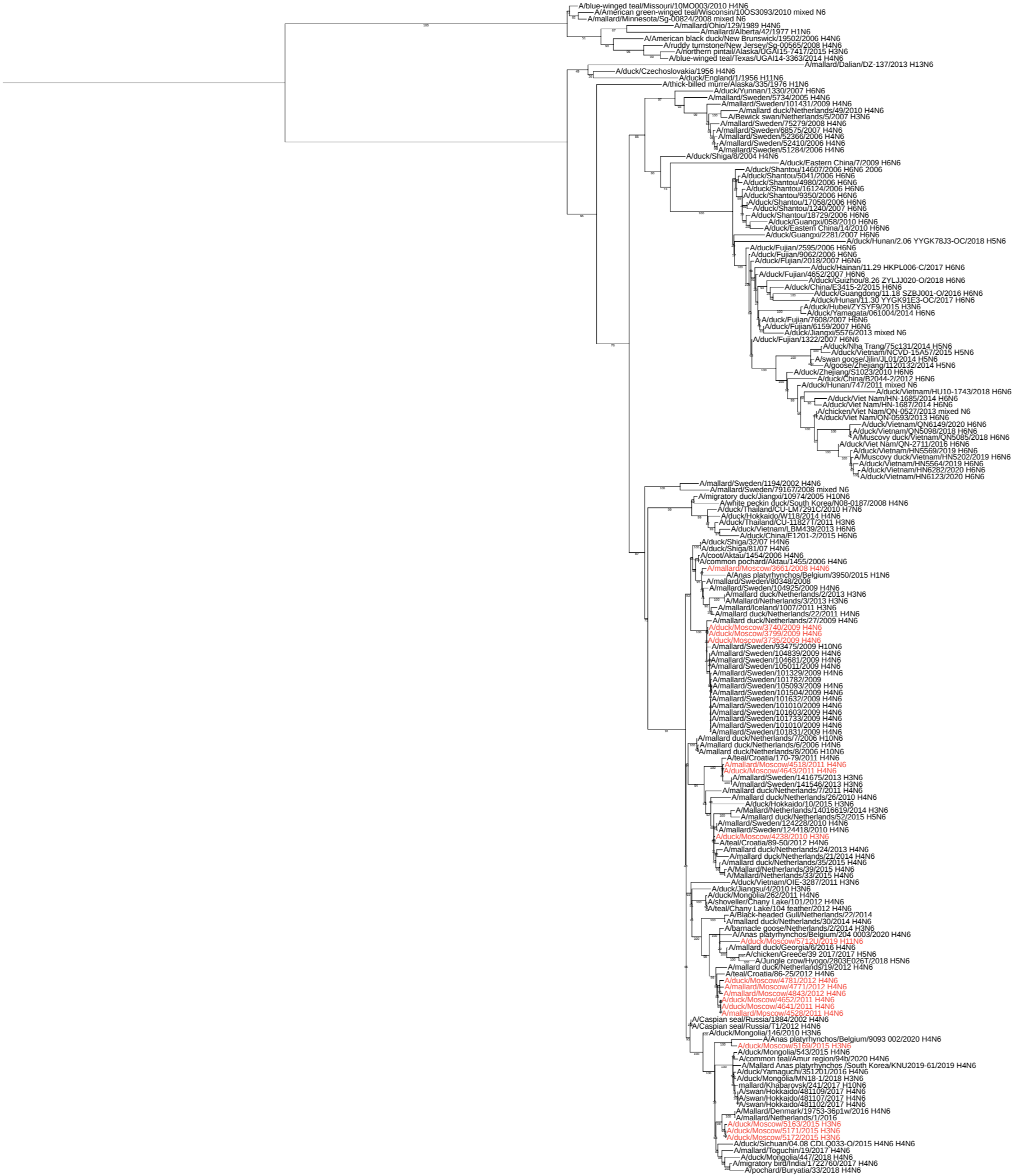

Supplement: Supplementary file 1 [file viruses-14-02624-s001.zip › Supplementary materials/figure S17 evolutionary tree of the N6 NA gene.pdf]

Tree scale: 0.1

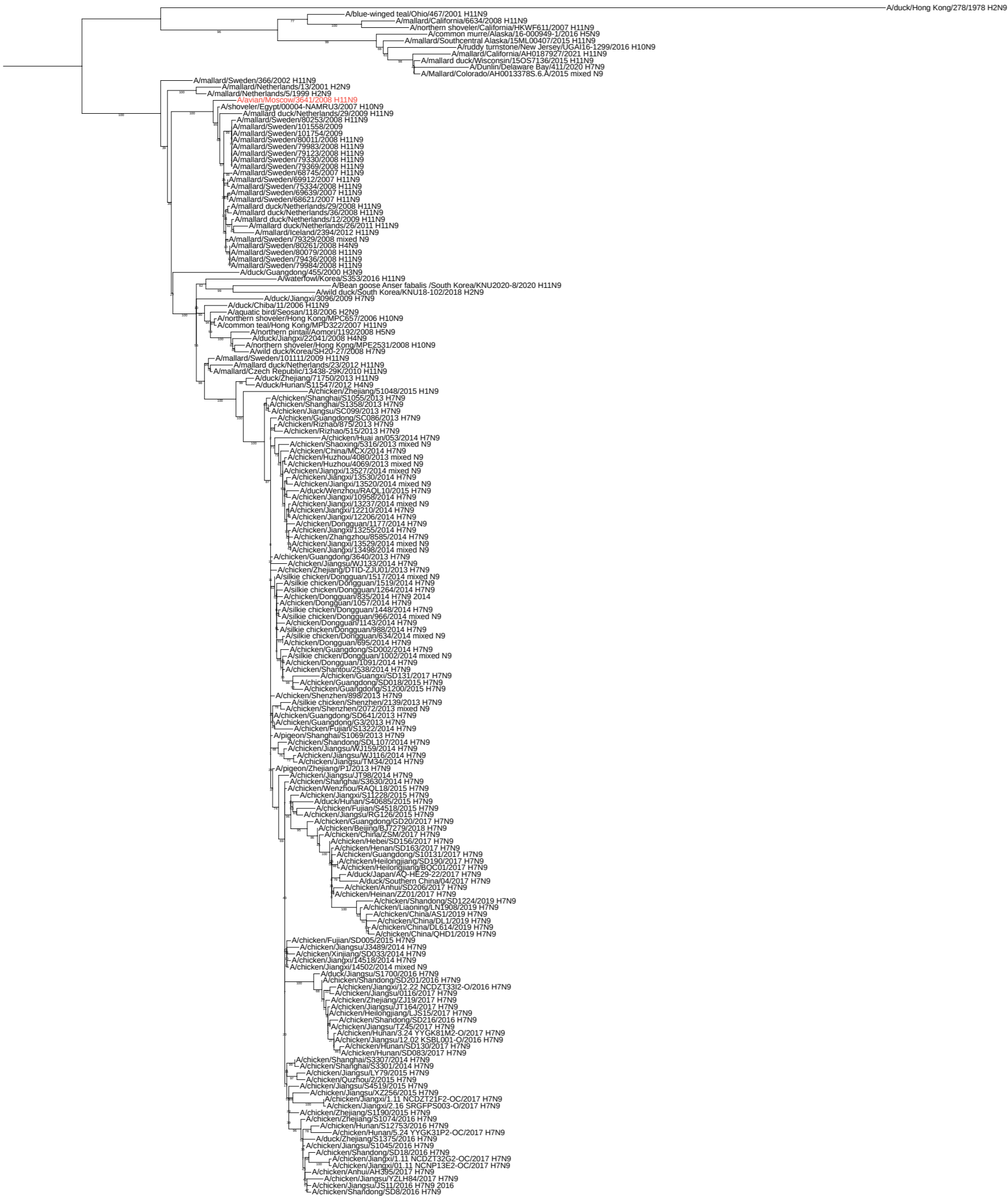

Supplement: Supplementary file 1 [file viruses-14-02624-s001.zip › Supplementary materials/figure S19 evolutionary three of the N9 NA gene.pdf]

Tree scale: 0.1

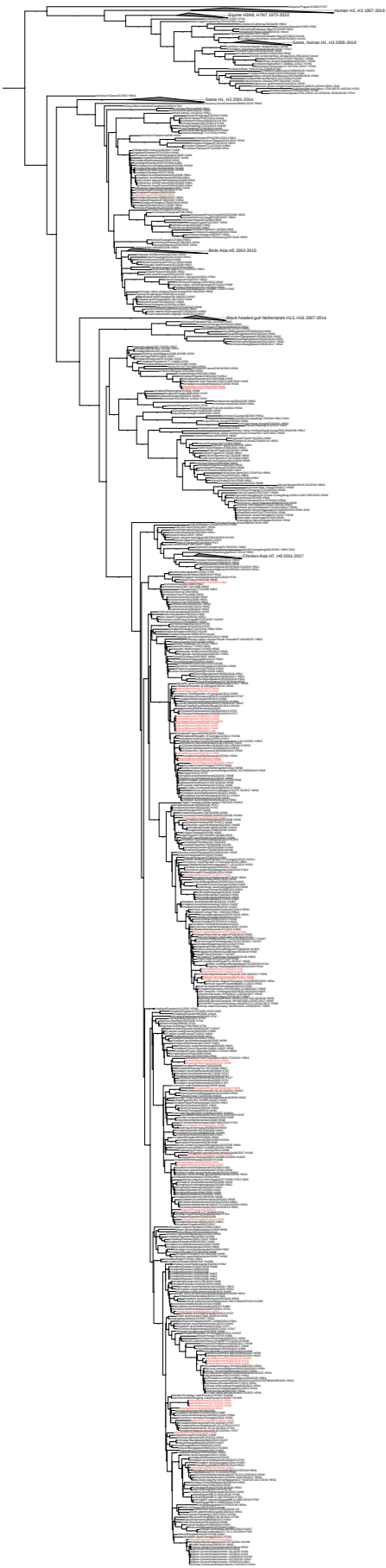

Supplement: Supplementary file 1 [file viruses-14-02624-s001.zip › Supplementary materials/figure S2 evolutionary tree of the PB2 gene.pdf]

Tree scale: 0.1

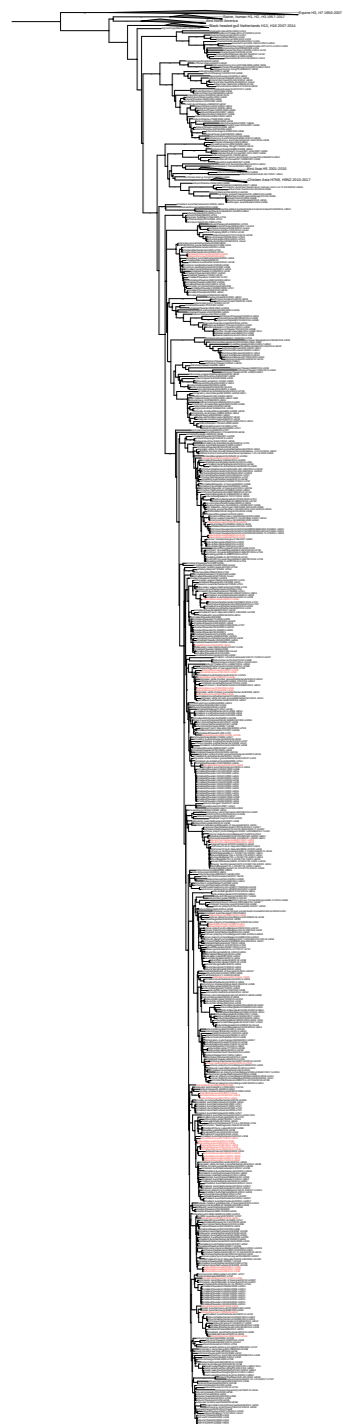

Supplement: Supplementary file 1 [file viruses-14-02624-s001.zip › Supplementary materials/figure S3 evolutionary tree of the PB1 gene.pdf]

Tree scale: 0.1

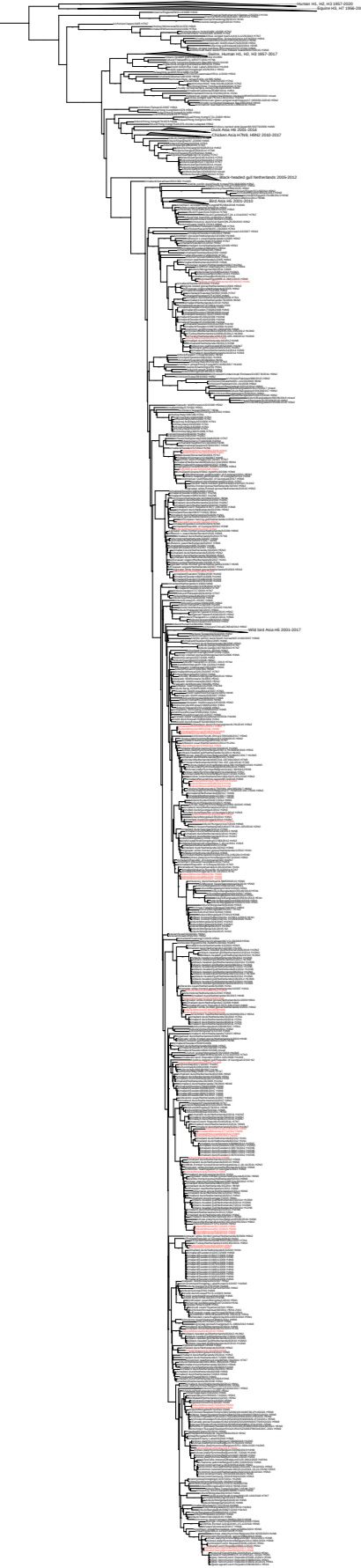

Supplement: Supplementary file 1 [file viruses-14-02624-s001.zip › Supplementary materials/figure S4 evolutionary tree of the PA gene.pdf]

$\sigma: 0.1$  

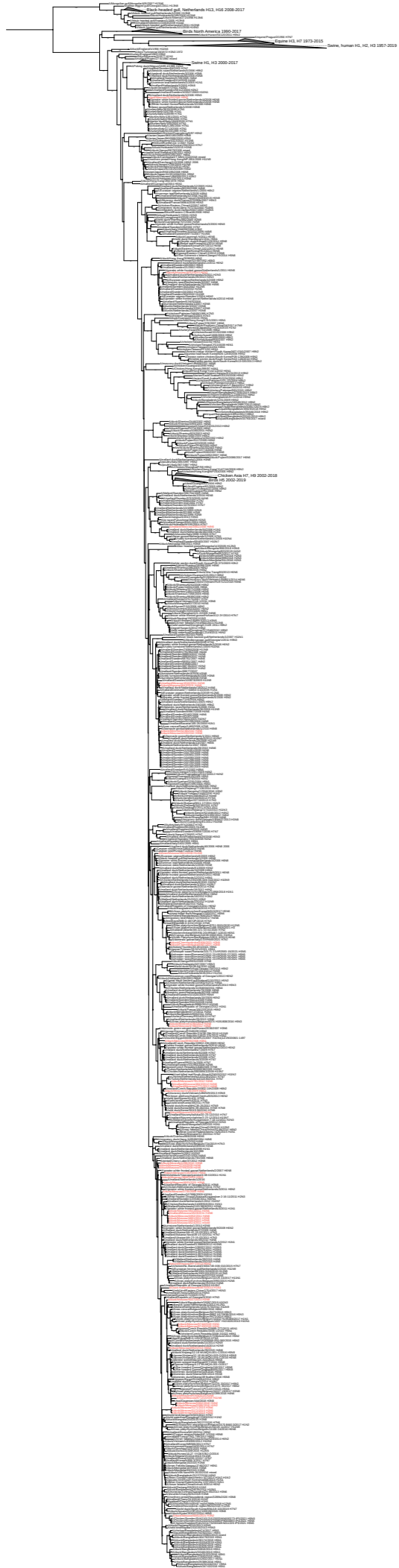

Supplement: Supplementary file 1 [file viruses-14-02624-s001.zip › Supplementary materials/figure S5 evolutionary tree of the NP gene.pdf]

Tree scale: 0.1

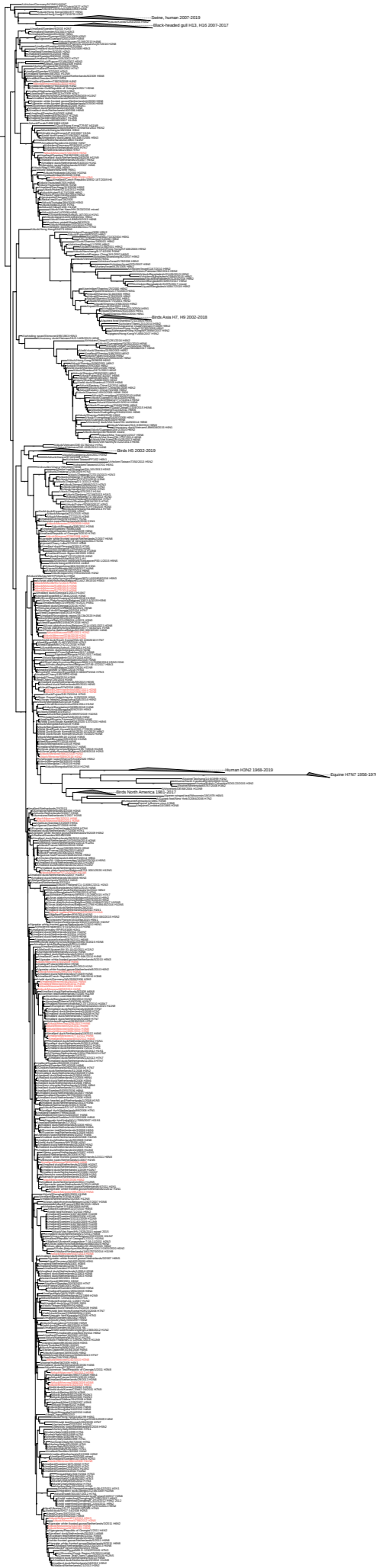

Supplement: Supplementary file 1 [file viruses-14-02624-s001.zip › Supplementary materials/figure S6 evolutionary tree of the M gene.pdf]

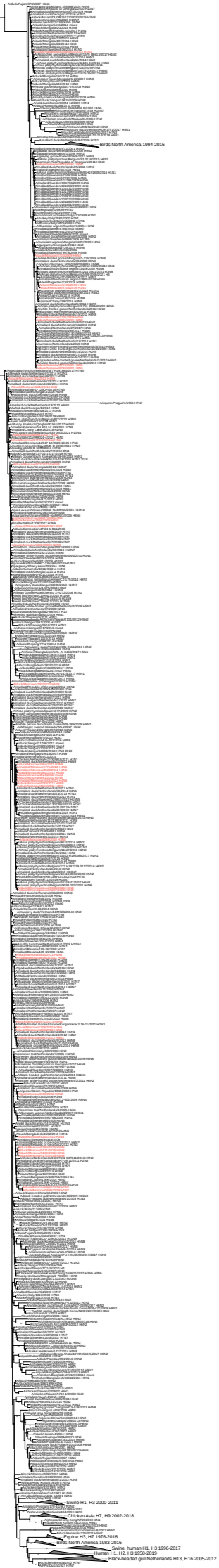

Supplement: Supplementary file 1 [file viruses-14-02624-s001.zip › Supplementary materials/figure S7 evolutionary tree of the NS gene.pdf]

Tree scale: 0.1

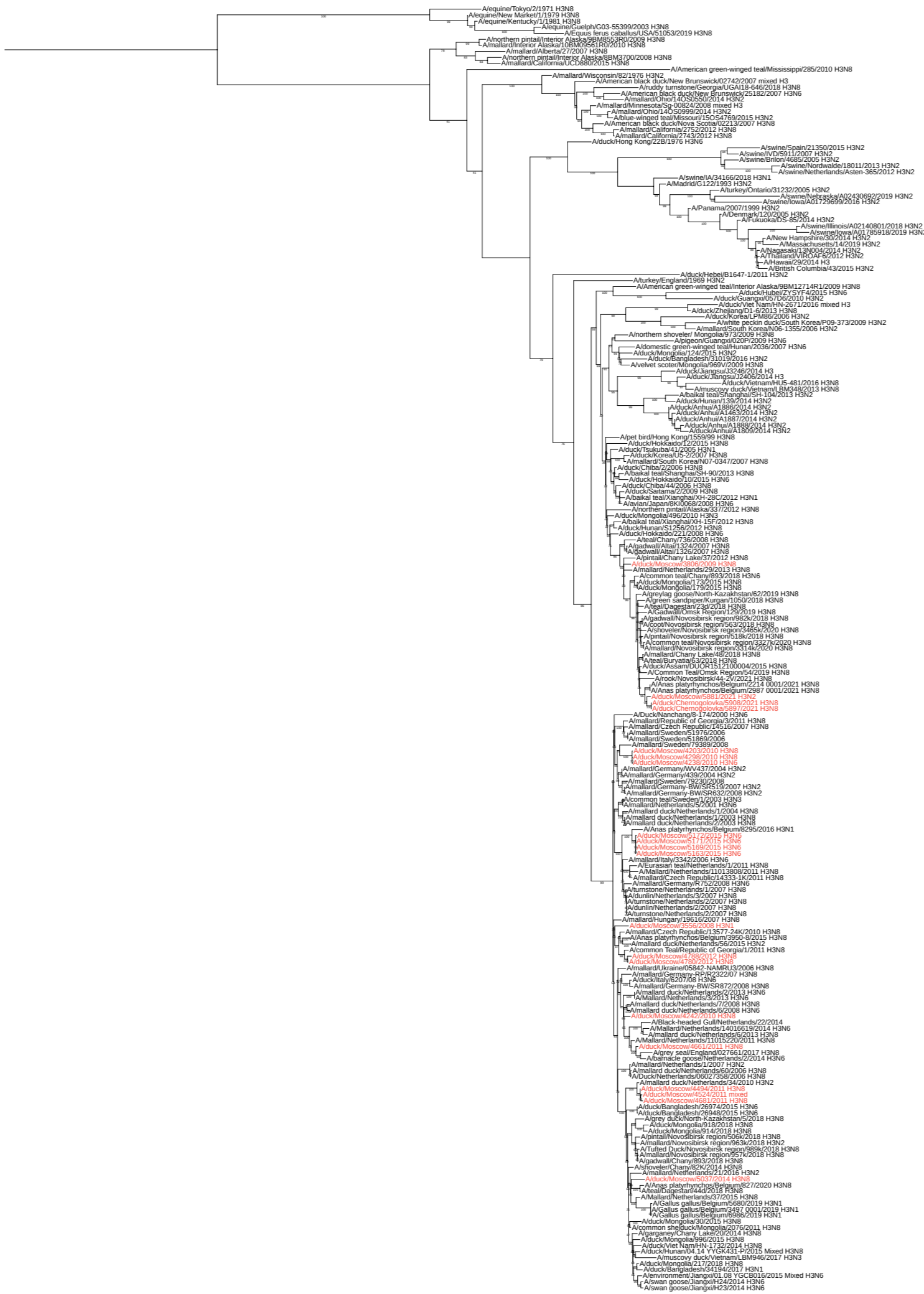

Supplement: Supplementary file 1 [file viruses-14-02624-s001.zip › Supplementary materials/figure S9 evolutionary tree of the H3 HA gene.pdf]
